# Mass and Energy Balances of Dry Thermophilic Anaerobic Digestion Treating Swine Manure Mixed with Rice Straw

**DOI:** 10.1155/2015/895015

**Published:** 2015-11-02

**Authors:** Sheng Zhou, Jining Zhang, Guoyan Zou, Shohei Riya, Masaaki Hosomi

**Affiliations:** ^1^Eco-Environmental Protection Research Institute, Shanghai Academy of Agricultural Sciences, Shanghai 201403, China; ^2^Shanghai Co-Elite Agricultural Sci-Tech (Group) Co., Ltd., Shanghai 201403, China; ^3^Institute of Engineering, Tokyo University of Agriculture & Technology, Tokyo 184-8588, Japan

## Abstract

To evaluate the feasibility of swine manure treatment by a proposed Dry Thermophilic Anaerobic Digestion (DT-AD) system, we evaluated the methane yield of swine manure treated using a DT-AD method with rice straw under different C/N ratios and solid retention time (SRT) and calculated the mass and energy balances when the DT-AD system is used for swine manure treatment from a model farm with 1000 pigs and the digested residue is used for forage rice production. A traditional swine manure treatment Oxidation Ditch system was used as the study control. The results suggest that methane yield using the proposed DT-AD system increased with a higher C/N ratio and shorter SRT. Correspondently, for the DT-AD system running with SRT of 80 days, the net energy yields for all treatments were negative, due to low biogas production and high heat loss of digestion tank. However, the biogas yield increased when the SRT was shortened to 40 days, and the generated energy was greater than consumed energy when C/N ratio was 20 : 1 and 30 : 1. The results suggest that with the correct optimization of C/N ratio and SRT, the proposed DT-AD system, followed by using digestate for forage rice production, can attain energy self-sufficiency.

## 1. Introduction

The number of scaled pig farms in Asia has greatly increased in recent years, while the disposal methods of swine manure are relatively underdeveloped. Particularly, China accounts for approximately 45–50% of the global pig production during the past decade [1, 2]. This has resulted in a significant increase in pig farm wastewater discharge, which has become an important source of water body pollution [3, 4]. In Japan, industrial treatment of swine waste is also becoming an important pathway for pig farm. During the process of industrial treatment of swine manure, the solids and liquid are first separated [5], followed by further treatment using both liquid and solid processes. This includes treating liquid phase using biological active sludge processes, and composting solid phase [6, 7]. However, some of the nutrients contained in swine manure cannot be recovered and are therefore discharged with the wastewater treatment plant effluent. Additionally, organic matter contained in the swine manure cannot be efficiently recovered as an energy source.

Anaerobic digestion is an efficient technology for livestock wastewater treatment, as well as an important technology for recovering biogas as a renewable energy source from organic substrates [8, 9]. Digestion of swine manure alone is usually unsuccessful because of its high ammonium concentrations and low C/N ratio [10–12]. As such, swine manure is preferably codigested with organic wastes containing high amounts of carbon, to improve the C/N ratio and to further increase biogas production. Codigestion of animal manure with various agroindustrial residues has been previously reported, with particular interest in the codigestion of animal manure with straws. Hills and Roberts [13] reported the benefits of codigesting plant material with low C/N animal manure. Specifically, they found that low C/N manure could provide a sufficient amount of nutrients, while the added plant materials with high carbon content could improve the C/N ratio and therefore decrease the risk of ammonia inhibition in the digestion process. Rice straw is one of the most important energy sources readily available in the rural areas in many countries, particularly in Asia, and it may also be used for biogas production through anaerobic digestion [5, 14]. In addition, dry anaerobic digestion is more beneficial than wet anaerobic digestion for compact reactors, because the process has lower water content and higher methane production [15, 16]. Furthermore, compared with mesophilic digestion processes, thermophilic digestion achieves higher rates of digestion and greater conversion of organic waste to gas [17, 18]. Therefore, a thermophilic digester can be loaded to a higher degree or operated at a lower solid retention time than at mesophilic conditions. But the thermophilic process temperature results in a higher risk for ammonia inhibition. Ammonia toxicity increases with increasing temperature [18].

China is one of the most abundant straw resources in the world, producing more than 620 million tons of straw in 2002, which is the most part of the biomass energy resources [19]. Rice production is also one of the most important agricultural production forms in Japan. In addition, besides common rice production, a series of forage rice varieties (*Oryza sativa* L.) have been developed for livestock feed in Japan. Some varieties have high levels of nonstructural carbohydrates in their stems and leaves [20], which can improve the digestibility of forage rice straw and enhance the biogas production of anaerobic codigestion of swine manure. Given this, we propose the following innovative system: swine manure produced on a pig farm is treated by the Dry Thermophilic Anaerobic Digestion (DT-AD) process with forage rice straw and generates biogas. Biogas is converted into heat and electricity through a Combined Heat and Power (CHP) system, which is used to run a DT-AD system. In addition, the thermophilic anaerobic digestion process is able to inactivate weed seeds, bacteria, viruses, fungi, and parasites in the feedstock which is of great importance when the digestate is used as fertilizer [21]. The best sanitation effect is obtained at thermophilic temperatures above 50°C and long retention times. In this study, the digested residue from the DT-AD process is used as fertilizer for forage rice production, and the grain is provided to pig farm as part of feed.

It is, of course, necessary to evaluate whether the proposed DT-AD system with forage rice production is energy self-supporting or not. Energy balance from swine manure discharge through the end-use of digestate and generated heat/power should be considered in the entire chain of the proposed DT-AD system. To evaluate the feasibility of swine manure treated by the proposed system, we engaged with the following study goals: (1) verify the methane yield of swine manure and rice straw treated by DT-AD method under different C/N ratios and solid retention time (SRT) and (2) calculate the mass and energy balances when the DT-AD system is used for swine manure treatment from a farm with 1000 pigs and the digested residue is used for forage rice production.

## 2. Material and Methods

### 2.1. Methane Production Assay

The straw of forage rice (*Oryza sativa* L. Takanari) was chopped into 20 mm pieces and then ground into small particles (Wonder Blender WB-1, Osaka Chemical Ltd. Co., Osaka, Japan), which were further sieved using 10-mesh sieve. Rice straw and swine manure were characterized in terms of their total solid (TS), volatile solid (VS), total nitrogen (TN), and total carbon (TC). As anticipated, rice straw was rich in organic matter and carbohydrates (VS = 82.3%; TC = 35.1%; TN = 0.43%), while swine manure had high nitrogen content (VS = 8.0%; TC = 4.35%; TN = 0.59%; NH_4_^+^-N = 2567 mg/kg). The inoculum used in this study was taken from a Dry Thermophilic Anaerobic Digestion pilot plant that treats solid garbage including kitchen garbage and office paper, which is run by Tokyo Gas and the Tokyo Environmental Public Service Corporation. To remove the degradable organic matter, the inoculum was incubated before the experiment without any added organic matter.

Samples with different C/N ratios (C/N = 10, 20, 30; named CN10, CN20, CN30 treatments), adjusted with swine manure and rice straw were designed to examine the improvement of anaerobic digestion at different treatment levels. Methane production assays were conducted as semicontinuous experiments in triplicate with 500-mL Duran laboratory bottles under dry thermophilic (55 degree Celsius) conditions. Biogas collection and substrate sampling was done every week. Biogas sample was taken with 50-mL syringes through the sampling tunnel of the stopper and then stored in a vacuumed vial (SVG-30, Nichiden-Rika Glass Co., Ltd., Hyogo, Japan). The volume of biogas produced in the Tetra Pak bag was also measured. The percentage of CH_4_ and CO_2_ in the biogas was measured using a GC-8A gas chromatograph (Shimadzu, Kyoto, Japan) equipped with a thermal conductivity detector and a 2-m stainless column packed with activated carbon (60/80-mesh sieve). The effects of different C/N ratios on methane yield were measured at SRT of 80 [13] and faster SRT of 40 days.

### 2.2. Mass and Energy Balances Calculation

**Figure 1:**
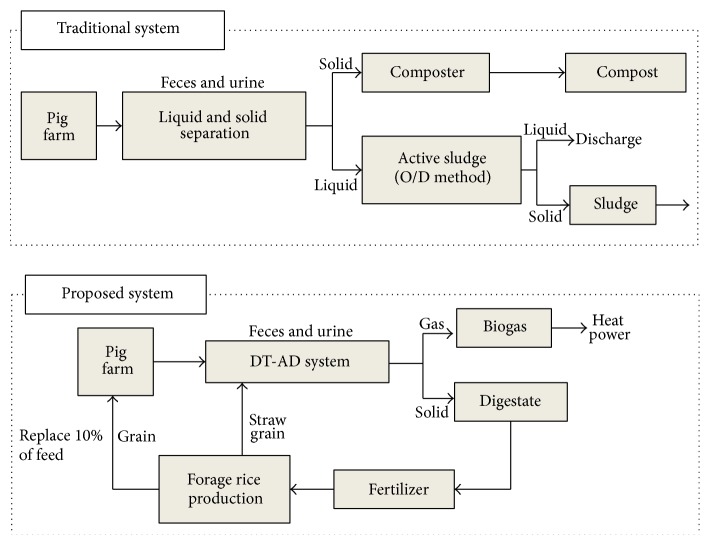
Calculation boundary of traditional and proposed systems.

For mass and energy balances calculations, a traditional swine manure treatment system was used as a control. This system includes wastewater treated using an Oxidation Ditch (O/D) system and solid waste composting, after the solid-liquid separation of swine manure. The entire system, including the treatment of swine manure using the proposed DT-AD system, followed by using digested residue as fertilizer for forage rice production, was calculated. The harvested grain was used as feed to support pig growth (replaced 10% of the total amount of feed). The mass balance was calculated by wet weight, in which water added by the process was included as an input. Evaporative water losses were not taken into account. The calculation of energy balance includes energy for DT-AD system operation and the energy invested in the DT-AD system plant construction. The calculation boundaries of traditional and proposed systems are shown in Figure 1, respectively.

#### 2.2.1. Traditional System

*Mass Balance Calculation Condition*. (1) Pig farm: the scale of the pig farm was designed as 1000 pigs; the basic unit of swine manure discharged from pig farm was 5.4 kg/pig/day, in which the biochemical oxygen demand (BOD) was 24352 mg/L, TN was 6759 mg/L, and *P* was 2722 mg/L. (2) Composter: the water content of solid phase was 72%; it was 62% after being adjusted with rice husk. The decomposition rate of the solid phase was 40%; (3) wastewater treatment: the BOD concentration of raw influent was 1200 mg/L and the MLSS of active sludge was 4000 mg/L. Removal efficiencies of BOD and TN were 96% and 80% [22], respectively. The settled sludge concentration was 12000 mg/L and the water content of concentrated sludge was 97%.

*Energy Balance Calculation Condition*. (1) Pig farm: the fuel, feed, and material used for one pig were converted into an energy unit; (2) composter: the fuel and material used for 1-ton raw material was converted into an energy unit; (3) the power and the material used for the wastewater treatment plant were converted into an energy unit.

#### 2.2.2. Proposed System

There were three parts of the mass energy balance calculation for the suggested system, which include running the pig farm, operating the DT-AD system, and producing the rice. The generated energy was calculated by converting biogas to power and heat using a Combined Heat and Power (CHP) system.

*Mass Balance Calculation Condition*. (1) Pig farm: swine manure (feces and urine) was discharged without solid and liquid separation processes; (2) the DT-AD system: the basic unit of biogas yield under different C/N ratios and SRTs are shown in Table 1; (3) the area of forage rice production depended on the nutrient content of digestate. The unit biomass of forage rice was 18.6 t/ha, which included 36% grain and 64% straw.

*Energy Balance Calculation Conditions*. (1) Pig farm: the grain of harvested forage rice replaced 10% of the total feed, so the energy equated with 10% of the feed was deducted from the total consumption energy; (2) the DT-AD system: biogas produced in the DT-AD system was converted into power and heat by CHP. CHP performances are shown in Table 2. The produced power and heat were provided to the DT-AD system, and the rest was sold for commercial purposes. The energy to run the DT-AD system included power, warming feedstock, and heat loss of the digestion tank and was based on the literature [20]. The energy required to raise the temperature of the feedstock and maintain the temperature of the heated tanks was calculated based on input volumes, tank dimensions, and insulation values; (3) the fuel and material used for forage rice production were converted into consumption energy, including heat, light, power, fertilizer, and farm machine. All energy calculation equations are shown in the appendices.

The term “net energy yield” is used for assessing the total system whether it is energy self-supporting or not. The “net energy yield” refers to the gross output energy minus the input energy in the entire chain of the proposed system as shown in

(1) $$\begin{array}{c} Net\;\;energy\;\;yield=Output\;\;energy-Input\;\;energy, \end{array}$$

where “output energy” was the heat and power produced by the DT-AD system; “input energy” for the proposed system was energy consumed in pig farm, DT-AD system, and rice production process, including power, fuel, feed, and material used in pig farm; energy for construction, digestion tank warming, heat loss from tanks, and electrical requirement in the DT-AD system; electrical and material requirement in the rice production process. On the other hand, there was no “output energy” in the traditional system. “Input energy” of traditional system has been described in the “energy balance calculation condition.”

## 3. Results and Discussion

### 3.1. Methane Yield under Different C/N Ratios and SRTs

**Figure 2:**
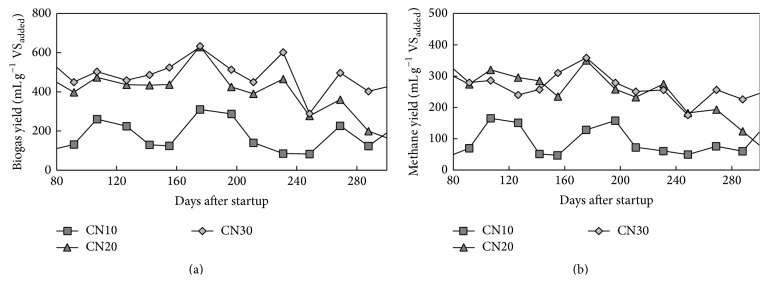
Biogas and methane yields under different C/N ratios under a SRT of 80 days.

Each treatment maintained a relatively stable biogas yield. Different levels of biogas were produced at different C/N ratios after 80 days following the start of the experiment, with an SRT of 80 days (Figure 2(a)). The average biogas yield at the CN10 treatment level was 177 ± 80 mL/g VS_added_ between 80 days and 300 days, which was half the yield of the other two CN treatments. Treatments with C/N ratios greater than 20 produced relatively high biogas yields, mostly greater than 400 mL/g VS_added_. When these higher levels are compared with CN10, the benefit of adding more rice straw to assist in anaerobic digestion of swine manure is clear, with increases up to approximately 2 and 3 times the average biogas production of CN20 (386 ± 139 mL/g VS_added_) and CN30 (474 ± 99 mL/g VS_added_). Biogas production in CN30 showed a higher and stable level, which is generally in agreement with the literature and indicates an optimal C/N ratio of 25–35 for rice straw [23, 24]. Conversely, biogas production of CN10 remained at an “inhibited steady state,” which is a condition where the process is stable, but with a low methane yield [10, 25], suggesting that the C/N ratio should be well balanced to avoid process failure by ammonia accumulation.

A similar trend is observed for the methane yield of each treatment, shown in Figure 2(b). Ammonium concentrations were significantly different among different C/N ratios. Particularly, the ammonium concentration in the CN10 treatment was above 4000 mg-N/kg, whereas, for the CN30 treatment, the ammonium level remained around 1500 mg-N/kg through the entire experimental period. Correspondently, the average methane yield was 91 ± 13 mL-CH_4_/g VS_added_ for CN10. The highest average methane yield was 265 ± 63 mL-CH_4_/g VS_added_ during the 80- to 300-day period with the CN30 treatment, followed by 252 ± 46 mL-CH_4_/g VS_added_ with the CN20 treatment. Methane yields for the CN20 and CN30 treatments, in which 22% and 35% of rice straw (resp.) were added to the feedstock, were similar to the results (213–269 mL-CH_4_/g VS_added_) of the codigestion of cow manure with 30% of crop (grass, sugar beet tops, and straw) in the feedstock [26]. The average methane content during the stable period ranged from 52% to 62% in the CN10, CN20, and CN30 treatments, which is similar to the methane content of biogas from typical lignocellulose materials (i.e., grass and maize silage) which range from 54% to 60% [25, 27].

The organic loading rate increased significantly when the SRT was reduced from 80 days to 40 days; these increases were from 3.2 kg-VS/m^3^/d to 6.4 kg-VS/m^3^/d. Consequently, the biogas yield increased significantly with SRT of 40 days under each C/N ratio, even though the trends of biogas productions were similar with those under SRT of 80 days. Finally, the biogas yield per VS was converted to biogas yield per ton of feedstock at different SRTs and C/N ratios for the mass and energy balances calculations shown in Table 1.

### 3.2. Comparison of Mass Balances between Traditional and Proposed Systems

**Figure 3:**
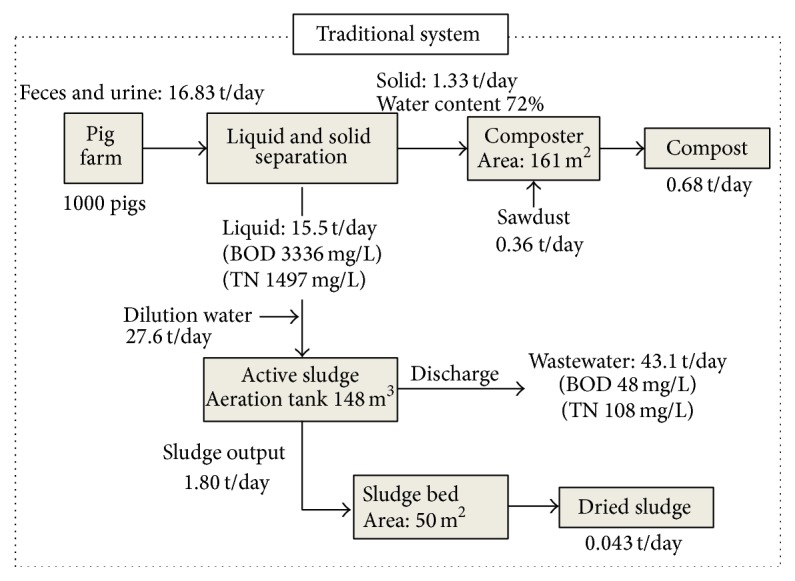
Mass balances for swine manure treatment using the traditional system.

The daily manure (feces and urine) discharged from 1000 pigs farm was 16.83 tons per day, which is the average amount of waste discharged from pig farm. In the mass balance of the traditional system (Figure 3), after solid and liquid separation, about 1.33 tons of solid phase per day flowed to the composter, while 15.5 tons of liquid phase flowed to the wastewater treatment plant. The BOD and TN concentration contained in the liquid phase was 3336 mg/L and 1497 mg/L, respectively. To treat the liquid phase by using active sludge, 27.6 tons per day of dilution water was added into the flow. Although the removal efficiencies of BOD and TN were relatively high, at 96% and 80%, the BOD and TN concentrations reached 48 and 108 mg/L. In fact, wastewater discharged from most of small scale pig farms cannot achieve these removal efficiencies. The TN concentration of effluent from the pig farm was higher, resulting from the special temporal effluent standard for effluent from livestock operations (700 mg-N/L from 2014) released by Ministry of the Environment, Japan, which is significantly higher than the uniform standard (100 mg-N/L) of industrial effluent. Besides the wastewater discharged from this system, sludge produced in the process of active sludge is another problem, because it must be disposed of and stored for dry treatment. Most of the sludge from active sludge process is dumped after dehydration (approximately 20% solid content) in Japan [15]. In this study, a space with 50 m^2^ was used for sludge drying process and produced 0.043 ton per day of dried sludge. Additionally, 0.68 ton per day of compost was also produced from solid phase after liquid and solid separation.

**Figure 4:**
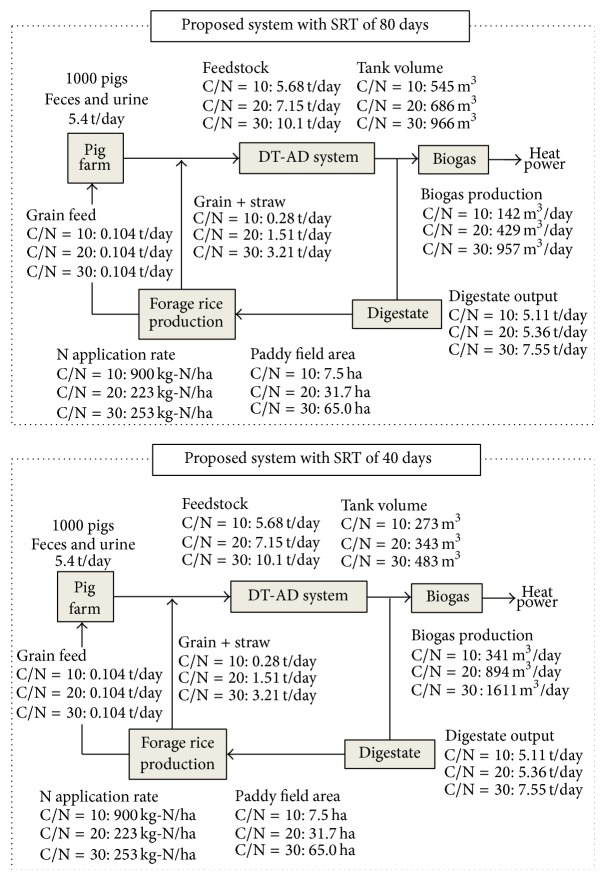
Mass balances for swine manure treatment using the proposed system under 80 and 40 days of SRTs.

Although the amount of swine manure discharged from 1000 pigs farm was the same, the volume of the digestion tank, biogas yield, digestate output, and paddy field area increased as the C/N ratio increased (Figure 4). To adjust the C/N ratio, different amount of straw and grain was added into swine manure for feedstock. The C/N ratio of 30 in feedstock needed the most amounts of grain and straw, which reached 3.21 tons per day whereas C/N ratio of 20 needed 1.51 tons per day. Correspondingly, the volume of digestion tank increased due to the amount of feedstock increasing. Meanwhile, the biogas production also increased with the loading rate of feedstock increasing. However, the volume of digestion tanks changed according to the different SRT even though the digestion was conducted under the same C/N ratio. Compared to the system with a SRT of 80 days at the same C/N ratio, the volume of digestion tank in the system with a SRT of 40 days was only half the size. The shorter SRT led to the higher organic loading rate. The organic loading rate increased from 3.2 kg-VS/m^3^/d to 6.4 kg-VS/m^3^/d with C/N ratio of 30. The higher organic loading rate resulted in higher biogas production, which could be converted into electricity and heat compared to the traditional system. Because the substrate must be mixed with digestate coming from the dry anaerobic digester before it is fed to the digester, organic loading rate can be allowed up to 10 kg-VS/m^3^/d, which is significantly higher than the loading rate of wet anaerobic digestion process (2–4 kg-VS/m^3^/d) [18]. Furthermore, in the proposed system including the DT-AD system, CHP, and forage rice production, no wastewater and sludge are discharged. Digestate could be used for forage rice production as fertilizer due to containing rich nutrients. Due to previous research, nutrients in livestock waste could be utilized by planting forage rice in paddy field and little nutrient discharged into environment [28, 29]. The area of paddy field was calculated basing on the required biomass amount for C/N ratio adjusts. It is obvious that the area was the highest when the biomass was used for C/N ratio of 30 adjusts. Part of harvested grain can be used for replacing the feed of 1000 pigs and all of the rest was used for C/N ratio adjusts.

### 3.3. Comparison of Energy Balances between Traditional and Proposed Systems

**Figure 5:**
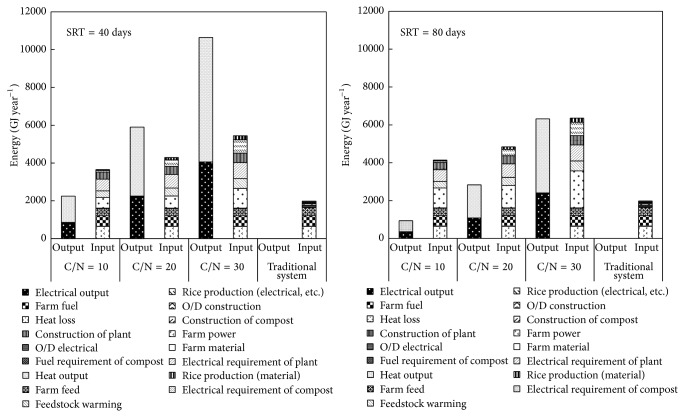
Comparison of energy balances between traditional and proposed systems under 80 and 40 days of SRTs.

Because the same scale of pig farms was used for the energy balances calculations comparing the traditional and proposed systems, the fuel and power consumed in these farms are both the same with the SRTs of 80 and 40 days (Figure 5). However, in the proposed system, 10% of the feed is replaced by grain that was fertilized using digestate, so the energy was deduced from the total consumption energy. Among the items contributing to energy consumption, heat loss from digestion tank in the DT-AD process was the largest element. The heat loss increased significantly with C/N ratio of feedstock increased at the same SRT because higher C/N ratio of feedstock made the tank bigger. Similarly, electrical requirement of DT-AD plant also increased with bigger tank. In addition, to achieve the higher C/N ratio, larger area of paddy field was needed for rice production, which resulted in the higher electrical demand for rice production for higher C/N ratio process. Furthermore, compared with the SRT of 80 days, energy consumption for the heat loss decreased significantly due to the smaller tank when the SRT was shortened from 80 days to 40 days.

The electrical and heat output increased not only at SRT of 80 days but at SRT of 40 days with C/N ratio increased, caused by the increasing biogas yield. However, although the biogas yield increased with C/N ratio increase, the net energy yield (Table 3) was negative for the proposed DT-AD system running with a SRT of 80 days, due to low biogas production and high heat loss. In contrast, the net energy yield with the SRT of 40 days was positive when the C/N ratios were 20 and 30, indicating that the generated energy was greater than the consumed energy by the entire system. The major reason was the biogas yield increased when the SRT was shortened to 40 days. Furthermore, the electric efficiency for energy balance calculation was 32% (Table 2). According to the literature, electric efficiencies of CHP up to 43% can be achieved, which can improve energy balance [18]. Compared to the proposed DT-AD system, the control scenario (a traditional pig farm with traditional O/D wastewater treatment) generated no energy but rather only consumed it for O/D wastewater treatment and electrical and fuel requirement of compost. Although the net energy yields under SRT of 80 days with C/N ratio as 30 as well as SRT of 40 days with C/N ratio as 10 were higher than that of traditional system, it is difficult to recognize the DT-AD system with these conditions as energy self-supporting systems due to negative net energy yield. However, the net energy yields under SRT of 40 days with C/N ratios as 20 and 30 were positive and significantly higher than that of traditional system, suggesting the DT-AD system with correct optimization can attain energy self-sufficiency.

## 4. Conclusion

Compared to the traditional pig farm with traditional wastewater treatment, there is no wastewater discharged from the suggested DT-AD system. Furthermore, it is possible to improve methane yield by adjusting the C/N ratio by adding rice straw into swine manure under dry thermophilic anaerobic conditions. For mass balance, in the proposed DT-AD system, biogas production increased as the C/N ratio increased. However, the area of forage rice vegetation also increased significantly. Because the scale of pig farms and the areas of paddy fields differ across regions, any proposed system must be considered in light of the regional characteristics of pig farms and paddy fields. On the other hand, the tank volume required for the proposed process can be reduced with a shorter SRT, suggesting that the area of the DT-AD plant can also be reduced.

With respect to energy balance, the consumption energy associated with the DT-AD system was dominant. The net energy yield was negative, due to low biogas production, when the DT-AD system was running at a C/N of 10 with SRTs of 80 and 40 days. The percentage of heat loss from the digestion tank with an SRT of 80 days was the highest, indicating that the DT-AD system cannot be energy self-supporting. However, the heat loss from the digestion tank decreased due to the compact nature of the tank when the SRT was reduced from 80 days to 40 days, while biogas production increased due to higher organic loading rate. The generated energy with the SRT of 40 days was greater than the consumed energy when the C/N ratios were 20 and 30, suggesting that, with the correct optimization, the DT-AD system can attain energy self-sufficiency.

## Figures and Tables

**Table 1:** Basic unit of biogas yield under different C/N ratios and SRTs.

	C/N = 10	C/N = 20	C/N = 30
Mixture (t-straw/manure)	0.052	0.28	0.594
Biogas yield at SRT of 80 days (Nm^3^/t)	25	60	95
Biogas yield at SRT of 40 days (Nm^3^/t)	60	125	160
Methane concentration	60	60	60
The percentage of volume reduction	10	25	25

**Table 2:** Performance of CHP plant.

	Parameter	Value
	Input methane concentration	60–70%
	Output (KW)	25
	Voltage (V)	200

Heat recycling	Recovery heat (KW)	40.6
	Temperature of water °C	70–75
	Recycle flow rate (L/min)	116

Efficiency	Electricity conversion efficiency (%)	32
	Heat recycle efficiency (%)	52
	General efficiency	84

**Table 3:** Net energy yield of proposed systems under 40 and 80 days of SRTs with different C/N ratios (GJ year^−1^).

	C/N = 10	C/N = 20	C/N = 30
SRT = 40	−1401	1610	5198
SRT = 80	−3204	−2009	−40

Net energy yield of traditional system was −1984 GJ year^−1^.

## Appendices

### A. Output Energy Calculation: Generated Energy

Consider the following:

(A.1) $$\begin{array}{c} EEO=BY\times MC\times Q_{net}\times CEE\times \frac{365}{10^{3}} \end{array}$$

EEO: Energy of electrical output (GJ year^−1^)
BY: Biogas yield (Nm^3^ day^−1^)
MC: Methane content (%)
*Q*_net_: Net calorific power of methane (MJ Nm^−3^)
CEE: Conversion efficiency of electric (%). Consider the following:
  (A.2) $$\begin{array}{c} EHO=BY\times Me\times Q_{net}\times CEH\times \frac{365}{10^{3}} \end{array}$$
EHO: Energy of heat output (GJ year^−1^)
BY: Biogas yield (Nm^3^ day^−1^)
Me: Methane content (%)
*Q*_net_: Net calorific power of methane (MJ Nm^−3^)
CEH: Recovery efficiency of heat (%).

### B. Input Energy Calculation: Consumed Energy

#### B.1. Pig Farm

Consider the following:

(B.1) $$\begin{array}{c} EEP=\frac{EP}{{CC}_{e}} \end{array}$$

EEP: Energy of electrical power requirement in* pig farm* with 1000 pigs (GJ year^−1^)
EP: Electrical requirement in* pig farm* with 1000 pigs (MWH year^−1^)
CC_e_: Conversion coefficient of electric (0.278). Consider the following:
  (B.2) $$\begin{array}{c} EFuP=\frac{\left(KR/CC_{K}+LR/{CC}_{L}\right)}{10^{3}} \end{array}$$
EFP: Energy of fuel requirement in* pig farm* with 1000 pigs (GJ year^−1^)
KR: Kerosene requirement in* pig farm* with 1000 pigs (L year^−1^)
LR: LPG requirement in* pig farm* with 1000 pigs (m^3^ year^−1^)
CC_K_: Conversion coefficient from Kerosene to energy (0.027248)
CC_L_: Conversion coefficient from LPG to energy (0.009488). Consider the following:
  (B.3) $$\begin{array}{c} EFeP=FC\times {CC}_{F}\times \frac{\left(1-R\right)}{10^{6}} \end{array}$$
EFeP: Energy of feed requirement in* pig farm* (GJ year^−1^)
FC: Feed cost for 1000 pigs per year (Yen year^−1^)
CC_F_: Conversion coefficient from feed to energy (21.37 kJ Yen^−1^)
*R*: Ratio of forage rice replace for feed (0.1). Consider the following:
  (B.4) $$\begin{array}{c} EMP=\frac{\left(D\times {CC}_{1}+R\times {CC}_{2}+B\times {CC}_{3}+Bu\times {CC}_{4}+C\times {CC}_{5}+AM\times {CC}_{6}+M\times CC_{7}\right)}{10^{6}} \end{array}$$
EMP: Energy of material requirement in* pig farm* with 1000 pigs (GJ year^−1^)
*D*: Drug cost for 1000 pigs per year (Yen year^−1^)
*R*: Rent cost for 1000 pigs per year (Yen year^−1^)
*B*: Breed cost for 1000 pigs per year (Yen year^−1^)
Bu: Building cost for 1000 pigs per year (Yen year^−1^)
AM: Agricultural machine cost for 1000 pigs per year (Yen year^−1^)
*M*: Management cost for 1000 pigs per year (Yen year^−1^)
CC_1–7_: Conversion coefficient from each item to energy (kJ Yen^−1^).

#### B.2. Compost Process (Only for Traditional System)

Consider the following:

(B.5) $$\begin{array}{c} EEC=\frac{EC}{{CC}_{e}} \end{array}$$

EEC: Energy of electrical requirement in* composter* (GJ year^−1^)
EC: Electrical requirement in* composter* (MWH year^−1^)
CC_e_: Conversion coefficient of electric (0.278). Consider the following:
  (B.6) $$\begin{array}{c} EFuC=\frac{LC/{CC}_{Lo}}{10^{3}} \end{array}$$
EFC: Energy of fuel requirement in* composter* (GJ year^−1^)
LC: Light oil requirement in* composter* (L year^−1^)
CC_Lo_: Conversion coefficient from light oil to energy (0.027248). Consider the following:
  (B.7) $$\begin{array}{c} ECC=UEc\times \frac{Yc/Ny}{10^{3}} \end{array}$$
ECC: Energy of construction for* composter* (GJ year^−1^)
UEc: Unit energy of construction for* composter* (MJ Yen^−1^)
Yc: Initiate construction cost for* composter* (Yen)
Ny: Service life (20 years).

#### B.3. Wastewater Treatment Process (Only for Traditional System)

Consider the following:

(B.8) $$\begin{array}{c} EEW=\frac{EW}{{CC}_{e}} \end{array}$$

EEW: Energy of electrical requirement in O/D* wastewater treatment* (GJ year^−1^)
EW: Electrical requirement in* wastewater treatment* (MWH year^−1^)
CC_e_: Conversion coefficient of electric (0.278). Consider the following:
  (B.9) $$\begin{array}{c} ECC=UEw\times \frac{Yw/Ny}{10^{3}} \end{array}$$
ECW: Energy of construction for O/D* wastewater treatment* (GJ year^−1^)
UE: Unit energy of construction for* wastewater treatment* (MJ Yen^−1^)
Yw: Initiate construction cost of* wastewater treatment plant* (Yen)
Ny: Service life (20 years).

#### B.4. DT-AD System Plant

Consider the following:

(B.10) $$\begin{array}{c} EHL=\frac{U}{10^{6}}\times 3600\times 24\times S\times \left(\;T-T_{a}\;\right)\times \frac{365}{10^{3}} \end{array}$$

EHL: Energy of heat lost from* digestion tank* (GJ year^−1^)
*U*: Overall heat transfer coefficient of* digestion tank* (W m^−2^ K^−1^)
*S*: Surface area of* digestion tank* (m^2^)
*T* and *T*_a_: Temperature of tank and air temperature (K). Consider the following:
  (B.11) $$\begin{array}{c} EHR=F\times C_{p}\times \left(\;T-T_{f}\;\right)\times \frac{365}{10^{3}} \end{array}$$
EHR: Energy of heat requirement for* feedstock warming* (GJ year^−1^)
*F*: Amount of feedstock input per day (t day^−1^)
*C*_*p*_: Specific heat coefficient (MJ t^−1^ K^−1^)
*T* and *T*_*f*_: Temperature of tank and feedstock temperature (K). Consider the following:
  (B.12) $$\begin{array}{c} EED=\frac{ER}{CC}\times \frac{365}{10^{3}} \end{array}$$
EED: Energy of electrical requirement in* DT-AD system* (GJ year^−1^)
ED: Electrical requirement per day in* DT-AD system* (KWH day^−1^)
CC: Conversion coefficient of energy (0.278). Consider the following:
  (B.13) $$\begin{array}{c} ECD=Y_{D}\times \frac{{UE}_{D}/Ny}{10^{3}} \end{array}$$
ECD: Energy of construction for* DT-AD system* (GJ year^−1^)
UE_*D*_: Unit energy of construction for* DT-AD system* (MJ Yen^−1^)
*Y*_*D*_: Initiate construction cost (Yen). Consider the following:
  (B.14) YD=1005.1×Xfeedstock  loading  rate+3565.1
Ny: Service life (20 years).

#### B.5. Rice Cultivation

Consider the following:

(B.15) EDR=LR×CCa+KR×CCb+GR×CCc+LO×CCd+MO×CCe+ERR×RA106

EDR: Energy of direct input in* rice cultivation* (GJ year^−1^)
LR: Light oil requirement in* rice cultivation* (L ha^−1^ year^−1^)
KR: Kerosene requirement in* rice cultivation* (L ha^−1^ year^−1^)
GR: Gasoline requirement in* rice cultivation* (L ha^−1^ year^−1^)
LO: Lubricant oil requirement in* rice cultivation* (L ha^−1^ year^−1^)
MO: Mixture oil requirement in* rice cultivation* (L ha^−1^ year^−1^)
ERR: Electrical requirement in* rice cultivation* (KJ ha^−1^ year^−1^)
RA: Rice field area needed for the system (ha)
CC_a–e_: Conversion coefficient from each item to energy (KJ L^−1^). Consider the following:
  (B.16) EIR=SC×CCI+AM×CCII+LIC×CCIII+BC×CCIV+CM×CCV+MC×CCVI×RA106
EIR: Energy of indirect input (material) in* rice cultivation* (GJ year^−1^)
SC: Seedling cost per ha (Yen ha^−1^ year^−1^)
AM: Agricultural material cost per ha (Yen ha^−1^ year^−1^)
LIC: Land and irrigation cost per ha (Yen ha^−1^ year^−1^)
BC: Building cost per ha (Yen ha^−1^ year^−1^)
CM: Car and agricultural machine cost per ha (Yen ha^−1^ year^−1^)
MC: Management cost per ha (Yen ha^−1^ year^−1^)
CC_I–VI_: Conversion coefficient from each item cost to energy (KJ Yen^−1^).
